# Differential innate immune responses of human macrophages and bronchial epithelial cells against *Talaromyces marneffei*

**DOI:** 10.1128/msphere.00258-22

**Published:** 2023-09-11

**Authors:** Yen-Pei Tan, Chi-Ching Tsang, Ka-Fai Chan, Siu-Leung Fung, Kin-Hang Kok, Susanna K. P. Lau, Patrick C. Y. Woo

**Affiliations:** 1 Department of Microbiology, School of Clinical Medicine, Li Ka Shing Faculty of Medicine, The University of Hong Kong, Pok Fu Lam, Hong Kong, China; 2 School of Medical and Health Sciences, Tung Wah College, Homantin, Hong Kong, China; 3 Tuberculosis and Chest Medicine Unit, Grantham Hospital, Aberdeen, Hong Kong, China; 4 Doctoral Program in Translational Medicine and Department of Life Sciences, National Chung Hsing University, Taichung, Taiwan; 5 The iEGG and Animal Biotechnology Research Center, National Chung Hsing University, Taichung, Taiwan; University of Georgia, Athens, Georgia, USA

**Keywords:** *Talaromyces marneffei*, human peripheral blood-derived macrophages, RNA sequencing, bronchial epithelial cells

## Abstract

*Talaromyces marneffei* is a thermally dimorphic fungal pathogen endemic in Southeast Asia. As inhalation of airborne conidia is believed as the major infection route, airway epithelial cells followed by pulmonary macrophages are the first cell types which the fungus encounters inside the host. In this study, we established an *in vitro* infection model based on human peripheral blood-derived macrophages (hPBDMs) cultured with the supplementation of autologous plasma. Using this model, we determined the transcriptomic changes of hPBDMs in response to *T. marneffei* infection by quantitative real-time reverse-transcription polymerase chain reaction as well as high-throughput RNA sequencing. Results showed that *T. marneffei* infection could activate hPBDMs to the M1-like phenotype and trigger a potent induction of chemokine and pro-inflammatory cytokine production as well as the expression of other immunoregulatory genes. In contrast to hPBDMs, there was no detectable innate cytokine response against *T. marneffei* in human bronchial epithelial cells (hBECs). Using a green fluorescent protein-tagged *T. marneffei* strain and confocal microscopy, internalization of the fungus by hBECs was confirmed. Live cell imaging further demonstrated that the infected cells exhibited normal cellular physiology, especially that the process of cell division could be observed. Moreover, *T. marneffei* also survived better inside hBECs than hPBDMs. Our results illustrated a potential role of hBECs to serve as reservoir cells for *T. marneffei* to evade immunosurveillance by phagocytes, from which the fungus reactivates when the host immunity is weakened and causes infection. Such immunoevasion and reactivation may also help explain the long incubation period observed for talaromycosis, in particular the travel-related cases.

**IMPORTANCE:**

*Talaromyces marneffei* is an important fungal pathogen especially in Southeast Asia. To understand the innate immune response to talaromycosis, a suitable infection model is needed. Here, we established an *in vitro T. marneffei* infection model using human peripheral blood-derived macrophages (hPBDMs). We then examined the transcriptomic changes of hPBDMs in response to *T. marneffei* infection with this model. We found that contact with *T. marneffei* could activate hPBDMs to the M1-like phenotype and induced mRNA expressions of five cytokines and eight immunoregulatory genes. Contrary to hPBDMs, such immunoresponse was not elicited in human bronchial epithelial cells (hBECs), despite normal physiology observed in infected cells. We also found that infected hBECs did not eliminate *T. marneffei* as efficiently as hPBDMs. Our observation suggested that hBECs may potentially serve as reservoir cells for *T. marneffei* to evade immunosurveillance. When the host immunity deteriorates later, then the fungus reactivates and causes infection.

## INTRODUCTION

*Talaromyces marneffei*, previously named as *Penicillium marneffei*, is the most important thermally dimorphic fungus causing respiratory, skin, and systemic mycosis in China and Southeast Asia, in particular northern Thailand and Vietnam (1). After its discovery in 1955, only 18 cases of human diseases were reported until 1985 (2). The appearance of the HIV pandemic saw the emergence of talaromycosis as an important opportunistic fungal infection in HIV-positive patients (3). Besides HIV-positive patients, *T. marneffei* infections have also been reported in other immunocompromised patients, such as transplant recipients, patients with systemic lupus erythematosus, those on corticosteroid therapy, with anti-interferon-gamma (anti-IFN-γ) autoantibody, and receiving anti-CD20 monoclonal antibodies or kinase inhibitors (4). Apart from China and Southeast Asia, there are imported talaromycosis cases in non-endemic countries (1). In addition, *T. marneffei* infections have been reported in patients from outside the area of endemicity who had no travel history to the endemic regions (5, 6). Since talaromycosis is associated with an unknown duration of incubation period, is often misdiagnosed in non-HIV-positive patients, possesses a high mortality rate if left untreated, and has a growing susceptible population, the etiological agent *T. marneffei* is regarded as one of the 10 most feared fungi in the world (7). With the availability of its draft genome in the past decade (8), a number of genes have been identified as responsible to the virulence of this fungus (9). Phylogenetically, *T. marneffei* is closely related to *Aspergillus fumigatus*, a filamentous fungus that causes highly fatal infections in immunocompromised patients.

*T. marneffei* grows in nature as a filamentous fungus. Similar to *A. fumigatus*, it is generally accepted that the route of *T. marneffei* infection is through inhalation of fungal conidia from the environment (10). In the human body, *T. marneffei* undergoes temperature-dependent dimorphic transition and becomes a yeast-like fungus (11). When the conidia are inhaled into the respiratory tract, the first cells that it encounters are the airway epithelial cells and pulmonary macrophages. However, unlike *A. fumigatus*, on which numerous host-pathogen interaction studies, including its interaction with human bronchial epithelial cells (hBECs) and macrophages, have been conducted to understand its pathogenesis, relatively little is known about the interaction between *T. marneffei* and human macrophages or airway epithelial cells and their associated host responses. In the literature, most of the few host-pathogen interaction studies of *T. marneffei* were performed using murine macrophages (12 – 34), while the use of human-derived macrophages for characterization has been limited (35, 36). However, significant differences between human and murine macrophages exist. For example, immunometabolism of human macrophages induced by lipopolysaccharides is different from that observed in murine macrophages, in which no glycolic reprogramming nor production of mitochondrial reactive oxygen species was observed in human macrophages (37). Most importantly, in those reports on *T. marneffei* pathogenesis, only a limited degree of cytokine induction, such as interleukin-1β (IL-1β), IL-8, and tumor necrosis factor (TNF) (38) which are similar to the pro-inflammatory response against *A. fumigatus* (39), can be observed in macrophages or macrophage cell lines that were infected with the conidia of *T. marneffei*, implying that further improvement of the current infection model would be crucial for in-depth study of early innate immune response against *T. marneffei*.

In this study, in order to understand the first steps of its pathogenesis process, we established an *in vitro* human macrophage model for the study of innate immune response to *T. marneffei* infection and compared it to those of *A. fumigatus*. Using high-throughput RNA sequencing (RNA-Seq), we then obtained a comprehensive global picture of human macrophage responses against *T. marneffei* infection. Furthermore, we examined the fate of *T. marneffei* when it interacts with hBECs and the differential cytokine responses as compared to macrophages.

## MATERIALS AND METHODS

### Fungal strains and culture conditions

Two fungal strains were characterized in this study. *T. marneffei* strain PM1, isolated from an HIV-negative patient suffering from culture-documented talaromycosis in Hong Kong, was grown on Sabouraud dextrose agar (SDA; Oxoid, Basingstoke, UK) containing 50 µg/mL of chloramphenicol (Calbiochem, San Diego, CA, USA) at room temperature. The second fungal strain *A. fumigatus* QC5096, obtained from the UK National External Quality Assessment Scheme originally isolated from an immunosuppressed patient with fatal cerebral abscess, was also grown on SDA containing 50 µg/mL of chloramphenicol but at 37°C and was used as an experimental control. For both fungal strains, after colony maturation (7 days for *T. marneffei* and 2–4 days for *A. fumigatus*), conidia were harvested in sterile distilled water and then stored at 4°C until use.

### Cell cultures

The murine macrophage cell lines J774A.1 (American Type Culture Collection [ATCC], Manassas, VA, USA) and RAW264.7 (ATCC) were cultured in Dulbecco’s modified Eagle medium (Gibco, Carlsbad, CA, USA) supplemented with heat-inactivated 10% fetal bovine serum (FBS; Gibco). hBECs were cultured in a combined medium containing one part of keratinocyte serum-free medium (Gibco) and one part of EpiLife medium (Gibco), which was further supplemented with 1% penicillin-streptomycin (Gibco). Isolation and culture of human peripheral blood-derived macrophages (hPBDMs) were performed as described below. All the cells were incubated at 37°C supplemented with 5% CO_2_.

### Preparation of hPBDMs

Preparation of hPBDMs was conducted according to Lee et al. (40) with modifications. Briefly, for each blood sample, blood fractionation was achieved by centrifugation at 1,000 *× g* for 15 min. The plasma obtained was heat-inactivated at 57°C for 30 min, and then cooled on ice for 10 min. Protein debris was then removed by centrifugation at 1,000 *× g* for 10 min, and the remaining precipitates were subsequently further removed by centrifugation at 13,000 *× g* for 2 min. The heat-inactivated autologous plasma was collected and stored at 4°C until use. As for the isolation of mononuclear cells, total blood cells were first diluted in RPMI 1640 medium (Gibco) supplemented with 2.5 mM of ethylenediaminetetraacetic acid (EDTA; Gibco) and then overlaid onto the Lymphoprep solution (Axis-Shield, Dundee, UK) in a ratio of 5:3 (vol/vol). Subsequently, the heterogeneous mixture was centrifuged at 1,800 *× g* for 30 min. Buffy coat was then collected at the interphase and resuspended in RPMI 1640 medium supplemented with 2.5 mM of EDTA. These cells were pelleted by centrifugation at 500 *× g* for 10 min and collected. Then, in order to lyse red blood cells, 0.8% (wt/vol) ammonium chloride (Stemcell Technologies, Vancouver, Canada) was added to the cells, followed by incubation on ice for 10 min. After several times of washing using phosphate-buffered saline (PBS; Oxoid) supplemented with 2.5 mM of EDTA, the remaining cells were resuspended in RPMI 1640 medium supplemented with 5% autologous plasma, transferred into three to five 10 cm tissue culture dishes (Techno Plastic Products [TPP], Trasadingen, Switzerland), and incubated at 37°C with 5% CO_2_. After incubation for at least 2 h, the cells were replenished with fresh RPMI 1640 medium supplemented with 5% autologous plasma. After an overnight incubation, the cells were chilled on ice for 10 min and were then collected in cold RPMI 1640 medium supplemented with 5 mM of EDTA by scraping. The collected cells were pelleted, resuspended in RPMI 1640 medium supplemented with 5% autologous plasma, and then seeded into the wells of six-well (at a density of 1 × 10^6^ cells/well) or 12-well (at a density of 5 × 10^4^ cells/well) tissue culture plates (TPP) for cytokine profiling and fungicidal activity studies, respectively. Following a further incubation at 37°C with 5% CO_2_ for 14 days, peripheral blood monocytes were allowed to differentiate into macrophages. Growth medium was replaced at 48- to 72-h intervals for cell maintenance.

### Cytokine profiling

hPBDMs were obtained as described above whereas J774A.1 cell line, RAW264.7 cell line, and hBECs were seeded into each well of a six-well tissue culture plate at a density of 1 × 10^6^ cells per well 1 day before infection. A total of 2 × 10^6^ *T. marneffei* PM1 or *A. fumigatus* QC5096 conidia, counted directly under the microscope using a hemocytometer, were added into each well of the cell culture plates and were allowed to co-incubate with the host cells at 37°C with 5% CO_2_ for different time points, including 0 h, 8 h, 24 h, and 48 h. Cells were then disrupted using QIAshredder (Qiagen, Hilden, Germany) and total nucleic acid extraction was conducted using the RNeasy Mini Kit (Qiagen) according to the manufacturer’s instructions. Subsequently, the total nucleic acids eluted in diethyl pyrocarbonate (DEPC)-treated water (Invitrogen, Carlsbad, CA, USA) were digested with DNase I (Ambion, Foster City, CA, USA) to remove genomic DNA, and finally, purified total cellular RNA was obtained. Extracted RNAs were used for reverse transcription using the SuperScript III Reverse Transcriptase (Invitrogen) with the oligo d(T) anchor primer 5´-GACCACGCGTATCGATGT CGACTTTTTTTTTTTTTTTTV-3´. Quantitative real-time reverse-transcription polymerase chain reaction (qRT-PCR) was performed using the Power SYBR Green Master Mix (Applied Biosystems, Foster City, CA, USA) according to the manufacturer’s instructions, with 1 µL of 10-fold diluted cDNA as the template for each reaction. The relative expressions of mRNAs were detected using primers as shown in Table S1. All samples were tested in triplicate. Data were normalized with respect to the glyceraldehyde-3-phosphate dehydrogenase gene (*GAPDH*) and the fold changes were calculated using the 2^−ΔΔCt^ method. Expression of cytokines were measured semi-quantitatively using the Proteome Profiler Human XL Cytokine Array Kit (R&D Systems, Minneapolis, MN, USA) according to the manufacturer’s instruction; and all samples were tested in duplicates.

### Library preparation and RNA-Seq

RNA samples extracted from hPBDMs infected with *T. marneffei* PM1 were also subjected to RNA-Seq. Purities of the samples were measured using NanoDrop 1,000 spectrometer (Thermo Fisher Scientific, Waltham, MA, USA). High-throughput Illumina sequencing was performed by the Centre for Genomic Sciences, Li Ka Shing Faculty of Medicine, The University of Hong Kong. Briefly, for each sample, a total of 700 ng of purified RNA were subjected to ribosomal RNA (rRNA) depletion using the Ribo-Zero Gold rRNA Removal Kit (Human/Mouse/Rat) (Illumina, San Diego, CA, USA). The cytoplasmic rRNA and mitochondrial rRNA were hybridized to biotinylated capture probes and then removed by washing. Subsequently, cDNA libraries were prepared using KAPA Stranded RNA-Seq Kit (KAPA Biosystems, Woburn, MA, USA) according to the manufacturer’s instructions. Theoretically, the rRNA-depleted RNAs were broken down into short fragments and used as templates for first-strand cDNA synthesis using random hexamer primer and reverse transcriptase. While during second-strand cDNA synthesis, the mRNA templates were removed, and replacement strands were generated to form blunt-end double-stranded (ds) cDNAs. These ds cDNAs were then subjected to 3´ adenylation and indexed adaptor ligation. The adaptor-ligated libraries were enriched by 14 cycles of PCR. The enriched libraries were then denatured and diluted to optimal concentrations and used in the cluster generation steps. Finally, HiSeq PE Cluster Kit v4 with cBot (Illumina) was used for cluster generation on the flow cell, whereas HiSeq SBS Kit v4 (Illumina) was used for pair-end 101 bp sequencing.

### Transcriptome analysis

Three independent biological replicates were included in the experiment. RNA-Seq data were analyzed using Tophat and Cufflinks (41). The protocol began with mapping raw sequencing reads to the human reference genome UCSC hg19 (available from the University of California Santa Cruz [UCSC] Genome Browser [42]) and a transcriptome assembly was produced. Differentially expressed or regulated genes and transcripts with statistical significance were then listed out with fragments per kilobase of transcript per million mapped reads (FPKM) as the expressional unit. Hereafter, genes with statistical significance (*P* < 0.001 and *q* < 0.05 as reported by Cuffdiff) were considered as differentially expressed (DEGs). A total of 452 DEGs were found across the biological replicates. The Gene Symbol ID for each DEG was retrieved from the UCSC database (https://genome.ucsc.edu/cgi-bin/hgTables). Subsequently, these DEGs with Gene Symbol IDs were used for heatmap generation using ClustVis (43). These genes were further tested in gene ontology (GO) enrichment analysis and gene functional clustering analysis using PANTHER (44) and mapped to Kyoto Encyclopedia of Genes and Genomes (KEGG) pathways by DAVID (45) with default settings.

### Fungicidal effect of host cells against *T. marneffei* live conidia

The test for intracellular conidia survival was conducted as previously reported (12, 13) with modifications. Briefly, hPBDMs were obtained as described above, whereas hBECs were seeded into each well of a 12-well tissue culture plate at a density of 5 × 10^4^ cells per well 1 day before infection. A total of 1 × 10^5^ *T. marneffei* PM1 conidia were added into each well of the tissue culture plates containing hPBDMs, hBECs, or the corresponding growth media only. These groups of cultures were allowed to incubate at 37°C with 5% CO_2_ for 24 h, followed by replenishment of their corresponding complete growth media supplemented with 240 U/mL of nystatin (Sigma-Aldrich, St. Louis, MO, USA). The cells were then harvested and lysed with 1% (wt/vol) Triton X-100 (Sigma-Aldrich) in PBS at days 1 and 2 post-infection and the cell lysates were collected for *T. marneffei* colony count. The fungal colony counts recovered from the day 1 cell lysates were considered as the baseline values for intracellular fungal survival analysis.

### Construction of green fluorescent protein (GFP)-tagged *T. marneffei* strain

The plasmid pAN7-1 (46) (a gift from Dr. P. J. Punt) containing the *A. nidulans gdp* gene promoter, hygromycin B-resistant gene, and *trpC* gene terminator was used to construct the GFP-tagged *T. marneffei* strain. Briefly, the enhanced GFP gene (*EGFP*) from pEGFP-C1 (Clontech Laboratories, Mountain View, CA, USA) was PCR-amplified using the primer pair LPW31624/LPW31625 (Table S1) and then cloned into pAN7-1 using the restriction enzyme *Bam*HI (New England Biolabs, Ipswich, MA, USA) (Fig. S2A). The resultant pAN7-1-GFP was then linearized with the restriction ezyme *Ahd*I (New England Biolabs) prior to fungal transformation into *T. marneffei* strain PM1 as described previously (13). The growth kinetics of the GFP-tagged *T. marneffei* PM1 (strain PM1-GFP) were measured for both mold and yeast forms to assess any phenotypic deviation from its parental strain (PM1).

### Imaging analysis

Imaging data were acquired using equipment maintained at the Faculty Core Facility of Li Ka Shing Faculty of Medicine, The University of Hong Kong. For fluorescence analysis, 1 × 10^6^ hBECs were first seeded on a sterile 24 mm × 32 mm coverslip in a 60 mm culture dish (TPP) 1 day before infection. A total of 1 × 10^7^ *T. marneffei* PM1-GFP conidia were used for the infection assay and the infected cells were harvested at 24 h and 48 h post-infection. Paraformaldehyde solution (4% wt/vol) was applied for cell fixation at room temperature for 10 min. After washing with PBS, the coverslips were carefully inverted on glass slides with mounting medium (Vectashield, Oakland, CA, USA) avoiding any air bubbles. The mounted samples were left in the dark overnight and finally secured using nail polish. Cells were then examined with the confocal microscope ZEISS LSM 710 (Carl Zeiss Microscopy, Jena, Germany) equipped with a 20× water or 40× oil immersion objective lens. Fluorescent images were further processed using ZEN 2.3 (Carl Zeiss Microscopy).

For live cell imaging, hBECs were seeded into the wells of six-well plates at a density of 1 × 10^5^ or 1 × 10^6^ cells per well 1 day before infection. After an overnight incubation, *T. marneffei* PM1 conidia were added into the wells at a multiplicity of infection (MOI) of 3 (3 × 10^5^ and 3 × 10^6^ colony-forming units per well, respectively). Live cell imaging was then performed 1 h after the addition of *T. marneffei* PM1 conidia. Images were captured every 5–10 min using the UltraVIEW ERS spinning disk system (PerkinElmer, Waltham, MA, USA) attached to the Axio Observer inverted microscope (Carl Zeiss Microscopy) with the Evolve 512 electron multiplying charge-coupled device camera (Teledyne Photometrics, Tucson, AZ, USA) for a 24-h incubation period. These images were further processed using MetaMorph 7.7.11 (Molecular Device, San Jose, CA, USA).

### Statistical analyses

Statistical calculations using the unpaired two-tailed *t*-test and analysis of variance was performed using Microsoft Excel (Microsoft, US) on gene expressions and fungal culture. Graphs were then extracted and further optimized for legibility using CorelDRAW Graphics Suite x7 (Alludo, Canada).

## RESULTS

### Establishment of an *in vitro* human macrophage system to study host-*T. marneffei* interactions

**Fig 1:**
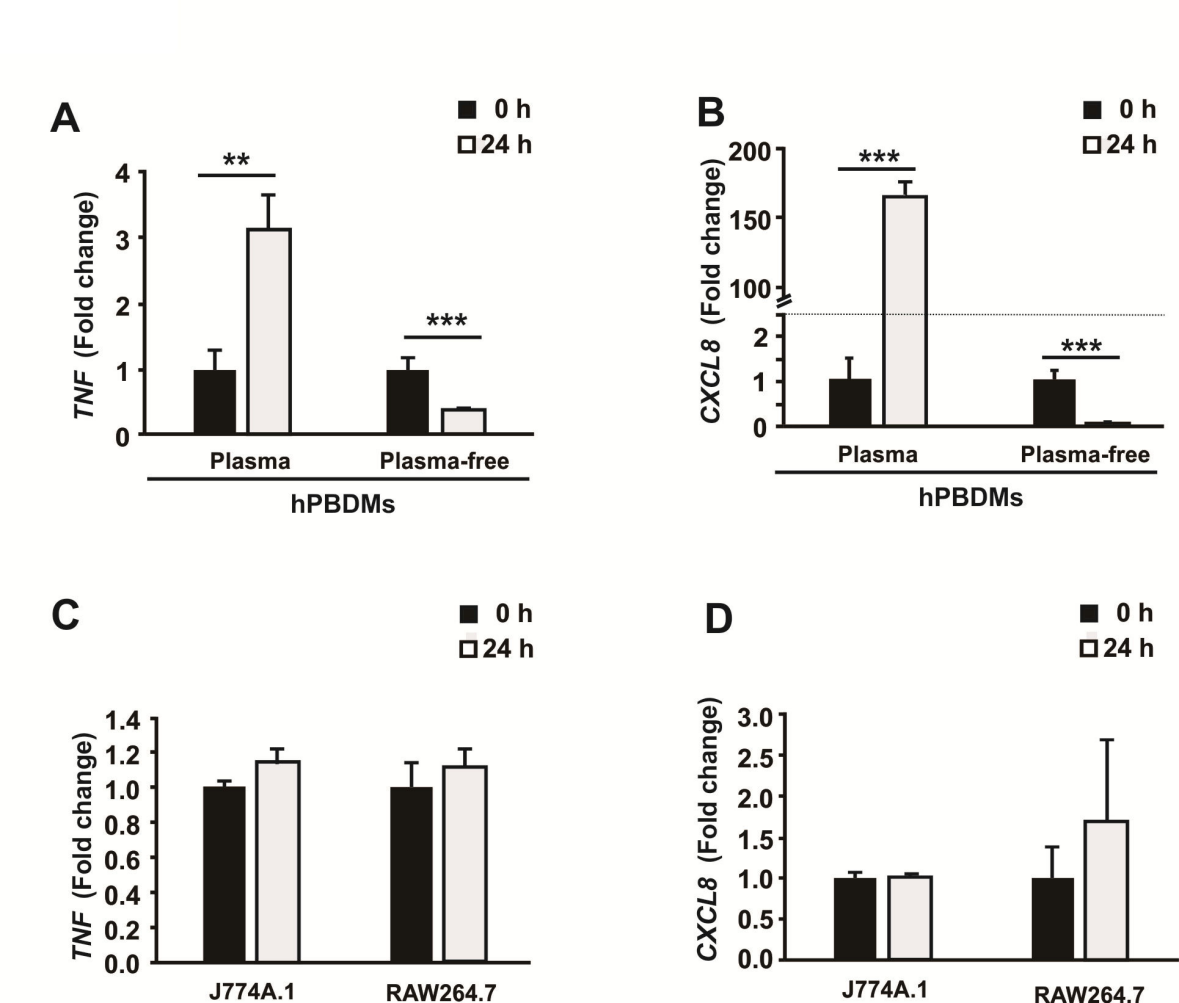
Cytokine responses of human and murine macrophages cultured in the presence or absence of plasma/serum upon *Talaromyces marneffei* infection. Upon *T. marneffei* infection, the relative expression of (**A**) *TNF* and (**B**) *CXCL8* mRNAs in hPBDMs could only be stimulated in the presence of autologous plasma; whereas no significant change in the mRNA expression of these two cytokines was observed when the culture medium was not supplemented with autologous plasma. The infection experiment was conducted in triplicate using blood cells isolated from three independent donors. The inability of *T. marneffei* to induce (**C**) *TNF* and (**D**) *CXCL8* mRNAs upon infection was also observed in the murine macrophage cell lines J774A.1 and RAW264.7. Data are presented as mean ± SD and comparisons were analyzed using unpaired *t*-test (two-tailed). **, *P* < 0.01; ***, *P* < 0.001.

*T. marneffei* could stay alive inside macrophages by unknown mechanisms (47). Although *in vitro* macrophage systems have already been widely applied to characterize the fungicidal capabilities of these phagocytic cells against *T. marneffei* (12, 13, 29), the intrinsic biological responses of macrophages upon encountering *T. marneffei* remain largely unknown. In order to develop a more physiological *in vitro* macrophage system for the study of interactions between the host and *T. marneffei*, we first characterized the innate immune responses of human and murine macrophages against *T. marneffei* live conidia by determining the mRNA expressions of the tumor necrosis factor gene (*TNF*) and C-X-C motif chemokine ligand 8 gene (*CXCL8*, also known as interleukin 8 gene [*IL8*]) during the early phase of infection. The experiment was designed to mimic natural infection by taking off general stimulants (e.g., lipopolysaccharide [LPS], IFN-γ, etc.) which had been used in previous studies (12, 13, 48). qRT-PCR revealed that upon *T. marneffei* infection, mRNA expressions of *TNF* and *CXCL8* were only significantly stimulated in hPBDMs when cultured with the supplementation of autologous plasma during the course of infection (Fig. 1A and B). In contrast, no significant difference in both the mRNA expressions of *TNF* and *CXCL8* before and after infection was detected in murine macrophage cell lines J774A.1 and RAW264.7 (Fig. 1C and D).

**Fig 2:**
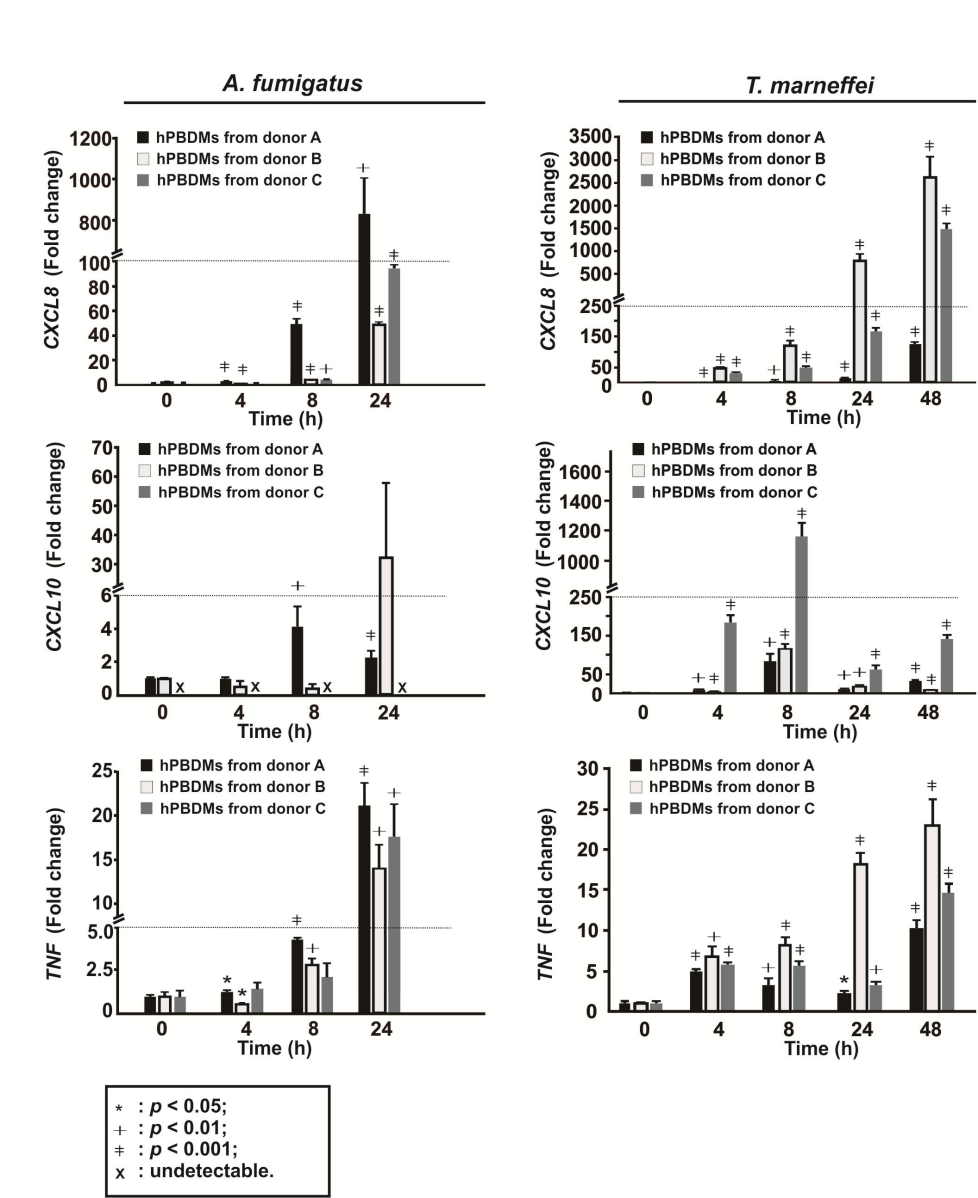
Changes of cytokine mRNA expression in hPBDMs upon *Aspergillus fumigatus* and *Talaromyces marneffei* infections. Relative mRNA expressions of *CXCL8* and *TNF* in hPBDMs infected with both fungi were significantly induced mainly after 4 h post-infection and the expression of *CXCL10* mRNA in hPBDMs was only significantly induced by *T. marneffei* infection, in which the expression level reached the peak at 8 h post-infection. The infection experiment was conducted in triplicate using blood cells isolated from three independent donors. The relative mRNA expression levels of *CXCL8*, *CXCL10*, and *TNF* were detected by qRT-PCR. Data are presented as mean ± SD and comparisons were analyzed using unpaired *t*-test (two-tailed). *, *P* < 0.05; †, *P* < 0.01; ‡, *P* < 0.001; ×, undetectable.

Subsequently, we examined the mRNA expression levels of these two cytokines as well as C-X-C motif chemokine ligand 10 gene (*CXCL10*, also known as IFN-γ-induced protein 10 gene [*IP-10*]) in hPBDMs infected with either *A. fumigatus* or *T. marneffei* live conidia. The results showed that mRNA expressions of *CXCL8* and *TNF* in hPBDMs infected with both fungi were significantly induced beginning from 4 h post-infection and the inductions were time-dependent (Fig. 2). However, the mRNA expression of *CXCL10* in hPBDMs was only significantly induced (>50-folds) by *T. marneffei* infection, which peaked at 8 h post-infection (Fig. 2).

To examine whether the upregulation in the mRNA expression of *CXCL8*, *CXCL10*, and *TNF* in hPBDMs infected with *T. marneffei* would indeed reflect changes in the expression of the corresponding cytokines, cytokine levels were measured semi-quantitatively using a multicytokine array. The results revealed that CXCL8 and CXCL10 cytokine expressions were significantly increased from 8 h post-infection while TNF cytokine level remained similar after infection (Fig. S1).

### Transcriptome profile of *T. marneffei*-infected hPBDMs and its functional annotation

**Fig 3:**
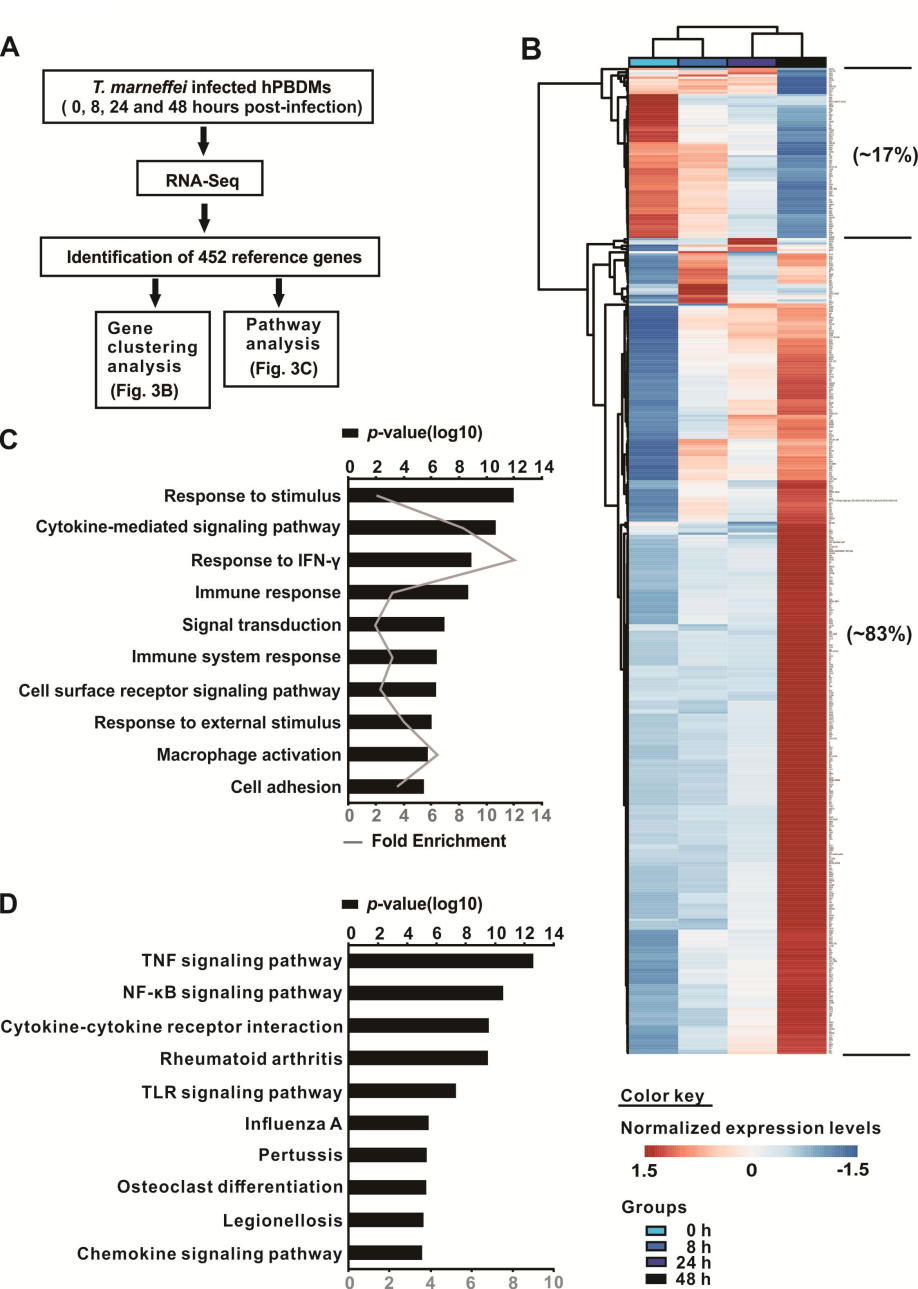
Transcriptional analysis of *Talaromyces marneffei*-infected hPBDMs. (**A**) A summary for the settings of the transcriptomic analysis in the present study. (**B**) Heat map of RNA-Seq transcriptome analysis for 452 significant DEGs. Detailed information regarding these 452 DEGs can be found in Table S2. (**C**) GO enrichment biological process analysis. The fold enrichment was obtained through comparing the background frequency of total genes falling under the same GO term. (**D**) KEGG pathway analysis. Both GO biological process analysis and KEGG pathway analyses revealed that these gene products were involved in host’s immune response. Both analyses only illustrate the top 10 pathways with the lowest *P*-values.

To characterize the global transcriptional responses of human macrophages upon *T. marneffei* infection, RNA samples from hPBDMs at 0 h, 8 h, 24 h, and 48 h post-infection were collected for RNA-Seq (Fig. 3A). Using the Illumina sequencing platform, each sample possessed an average throughput of 4.2 Gb. In terms of sequence quality, an average of 93% bases achieved a quality score of Q30, which denoted a 99.9% accuracy of a base call. The raw data were further analyzed using Tophat and Cufflinks (41). The resulting differentially expressed genes were further analyzed using different bioinformatic tools. A total of 452 genes were found significantly up- or down-regulated during the infection course. ClustVis analysis showed that 375 DEGs (~83.0%) were up-regulated while the remaining 77 DEGs (~17.0%) were down-regulated (Fig. 3B). GO biological process enrichment analysis revealed that these DEGs were most likely involved in immune processes, such as cytokine-mediated signaling pathway, response to IFN-γ and macrophage activation, etc. (Fig. 3C). Similarly, KEGG pathways analysis also revealed that different immune pathways, including TNF signaling pathway, NF-κB signaling pathway, cytokine-cytokine receptor interaction, etc., were involved (Fig. 3D). Moreover, a total of 25 genes associated with macrophage activation were also found differentially expressed in our RNA-Seq data (Table 1).

**TABLE 1:** Differentially expressed genes related to macrophage activation

Differentially expressed gene	Gene symbol	M1:M2 ratio^*a*^	Expression level at different time points post-infection (FPKM)^*b*^				Expression pattern post-infection^*c*^
			0 h	8 h	24 h	48 h	
Membrane receptors							
C-C motif chemokine receptor 7	*CCR7*	+	0.33	1.23	1.29	4.31	↑
Interleukin 15 receptor subunit alpha	*IL15RA*	+	1.05	3.51	3.13	4.18	↑
Interleukin 7 receptor	*IL7R*	+	0.70	1.41	1.62	5.57	↑
Membrane spanning 4-domains A6A	*MS4A6A*	−	9.14	5.81	4.76	1.85	↓
Membrane spanning 4-domains A4A	*MS4A4A*	−	119.44	105.33	93.98	76.35	↓
Cytokines and chemokines							
C-X-C motif chemokine ligand 10 (IFN-γ-inducible protein 10)	*CXCL10*	+	0.14	37.71	3.86	6.44	↑
Tumor necrosis factor	*TNF*	+	0.68	2.52	2.93	6.04	↑
C-C motif chemokine ligand 5	*CCL5*	+	2.16	2.44	3.11	6.18	↑
C-C motif chemokine ligand 20	*CCL20*	+	0.39	7.58	8.02	40.53	↑
Apoptosis-related genes							
Baculoviral IAP repeat containing 3	*BIRC3*	+	7.64	18.45	13.77	14.38	↑
Solute carriers							
Solute carrier family 2 member 6	*SLC2A6*	+	4.88	22.14	29.39	34.72	↑
Enzymes							
Hydroxysteroid 11-beta dehydrogenase 1	*HSD11B1*	+	11.94	16.38	22.76	25.49	↑
Proteasome activator subunit 2	*PSME2*	+	58.57	82.96	109.52	123.42	↑
Proteasome subunit beta 9	*PSMB9*	+	21.18	32.60	34.47	40.60	↑
Extracellular mediators							
Apolipoprotein L3	*APOL3*	+	4.87	27.94	19.37	28.36	↑
Apolipoprotein L1	*APOL1*	+	7.50	10.08	10.54	12.14	↑
Endothelin 1	*EDN1*	+	0.03	0.19	0.61	5.33	↑
Inhibin subunit beta A	*INHBA*	+	0.30	0.67	1.87	12.01	↑
Apolipoprotein L6	*APOL6*	+	19.06	36.15	28.61	35.58	↑
Transforming growth factor beta induced	*TGFBI*	−	29.93	25.94	21.53	11.68	↓
Selenoprotein P	*SELENOP* (*SEPP1*)	−	65.93	48.15	22.10	9.27	↓
Fibronectin 1	*FN1*	−	322.14	281.24	198.96	148.86	↓
Fibrinogen-like 2	*FGL2*	−	33.66	34.99	33.97	22.49	↓
DNA-binding factors							
Interferon regulatory factor 1	*IRF1*	+	7.71	35.29	15.48	27.24	↑
MAF bZIP transcription factor	*MAF*	−	19.18	16.85	14.76	11.74	↓

^*a*^ Differential gene expression in activated macrophages (M1 versus M2 polarization) reported by Martinez et al. (49). +, expression level in M1-like phenotype > that in M2-like phenotype; −, expression level in M1-like phenotype < that in M2-like phenotype.

^*b*^ FPKM, fragments per kilobase of transcript per million mapped reads.

^*c*^ ↑, up-regulated; ↓, down-regulated.

**Fig 4:**
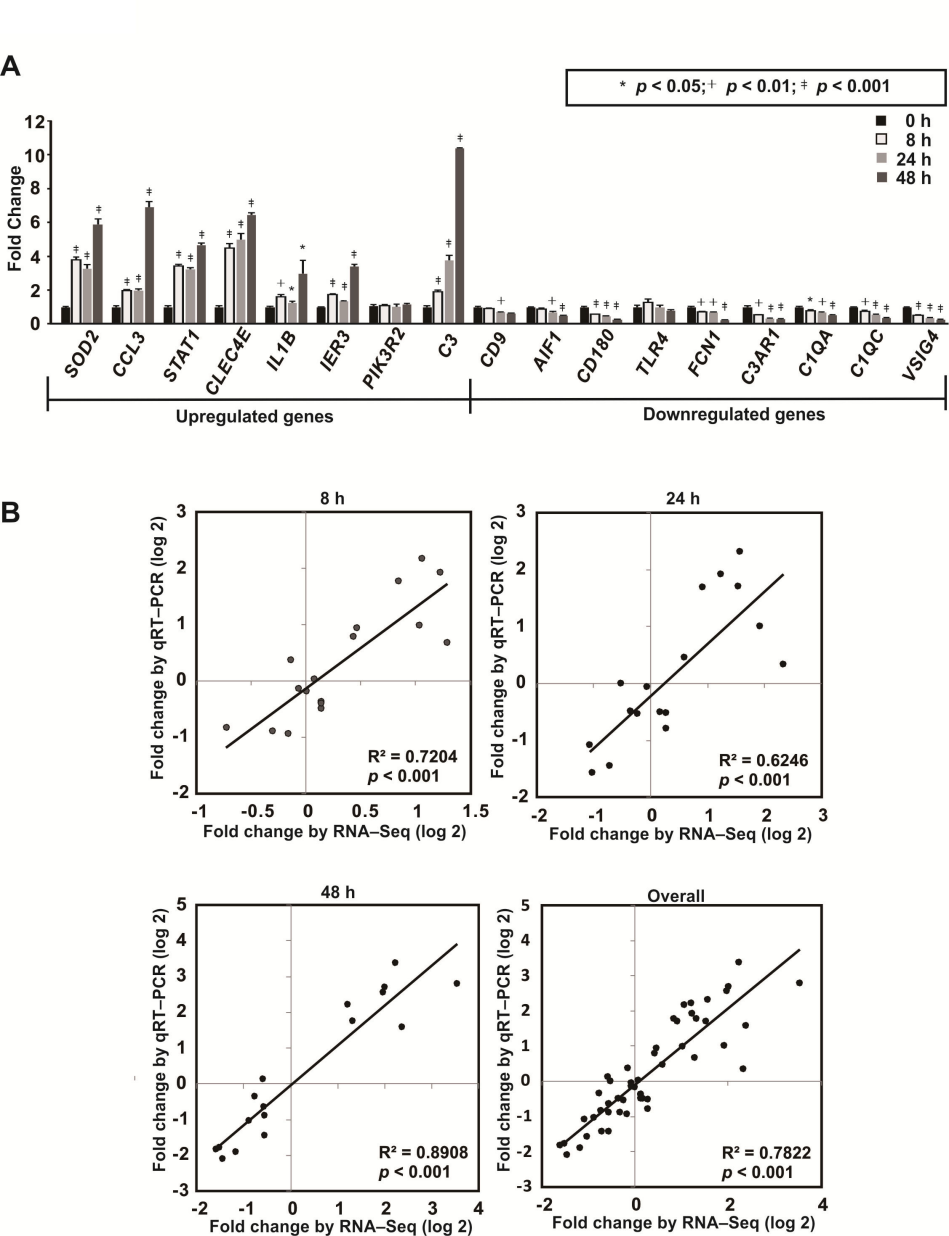
Validation of RNA-Seq data by qRT-PCR. (**A**) qRT-PCR results of the 17 DEGs (*SOD2*, *CCL3*, *STAT1*, *CLEC4A*, *IL1B*, *IER3*, *PIK3R2*, *C3*, *CD9*, *AIF1*, *CD180*, *TLR4*, *FCN1*, *C3AR1*, *C1QA*, *C1QC,* and *VSIG4*). Data are presented as mean ± SD and comparisons were analyzed using unpaired *t*-test (two-tailed). *, *P* < 0.05; †, *P* < 0.01; ‡, *P* < 0.001. (**B**) The correlation of RNA-Seq and qRT-PCR was further calculated by modeling the relationship of fold change obtained by RNA-Seq (x-axis) and data obtained using qRT-PCR (y-axis).

Since qRT-PCR has been considered one of the most promising approaches for gene expression analysis (50), we conducted qRT-PCR to verify our RNA-Seq data. The expression of a total of 17 genes (*SOD2*, *CCL3*, *STAT1*, *CLEC4A*, *IL1B*, *IER3*, *PIK3R2*, *C3*, *CD9*, *AIF1*, *CD180*, *TLR4*, *FCN1*, *C3AR1*, *C1QA*, *C1QC,* and *VSIG4*), which have been reported to play roles in innate immune response (51), were further examined (Fig. 4A). The correlation of the RNA-Seq data with the qRT-PCR data was analyzed using the log_2_ fold change measurement of the DEGs for linear regression analysis. The results showed that the *R^2^* values for the mRNA expression levels at 8 h, 24 h, and 48 h post-infection determined by the two approaches were 0.7204, 0.6246, and 0.8908, respectively (Fig. 4B). In general, the expressions of these DEGs were considerably similar as revealed by both RNA-Seq and qRT-PCR (*R^2^* = 0.7822, *P <* 0.001).

### Immunological silencing of *T. marneffei*-hBECs

**Fig 5:**
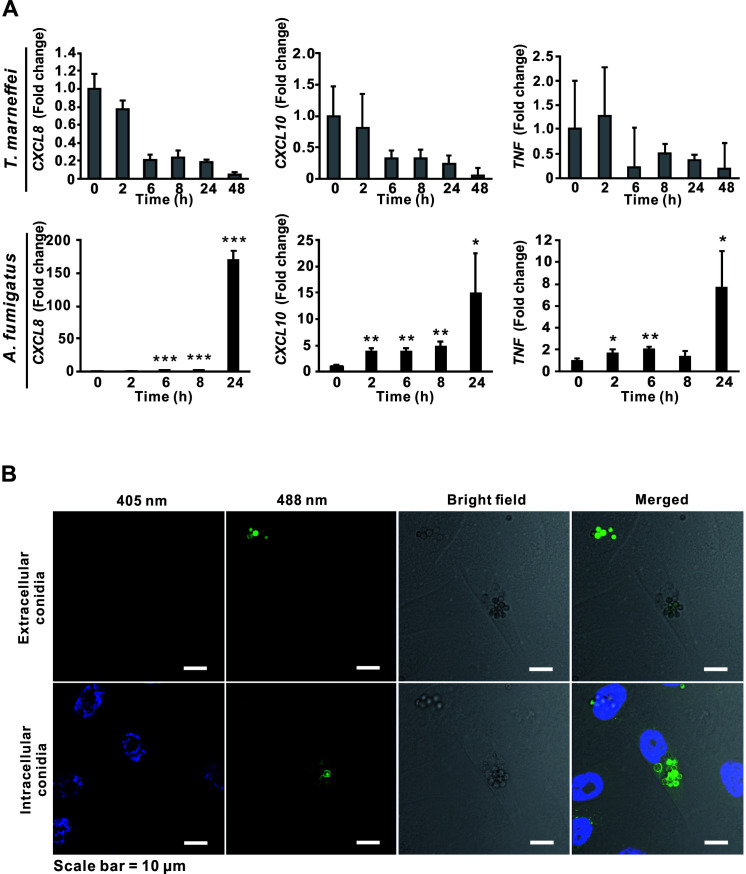
Immunological silencing of *Talaromyces marneffei-*infected hBECs. (**A**) The relative mRNA expression levels of *CXCL8*, *CXCL10,* and *TNF* were detected by qRT-PCR. Data are presented as mean ± SD and comparisons were analyzed using unpaired *t*-test (two-tailed). *, *P* < 0.05; **, *P* < 0.01; ***, *P* < 0.001 versus samples collected at 0 h post-infection. (**B**) The distribution of extracellular and intracellular conidia of GFP-tagged *T. marneffei* in hBECs.

Airway epithelial cells are believed to serve as a physical barrier to block pathogens, and recently, their roles in immune response have been demonstrated (52). The fact that *A. fumigatus* conidia could be taken up by airway epithelial cells (53, 54) prompted further examination of the interactions between airway epithelial cells and other fungal pathogens, such as *T. marneffei*. However, in the current study, it was demonstrated that unlike the situation observed for *A. fumigatus* infection in hBECs where the mRNA expressions of *CXCL8*, *CXCL10*, and *TNF* during the early infection stage (within the first 24 h post-infection) in the host cells were markedly up-regulated, no significant change in the mRNA expressions of these cytokines was detected in hBECs upon encountering *T. marneffei* live conidia (Fig. 5A). Moreover, the induction of interleukin 1 beta (*IL1B*) and interleukin 6 (*IL6*), which are proinflammatory cytokines related to nucleotide-binding oligomerization domain-like receptor family pyrin domain containing 3 inflammasome (55), was also not observed; while the mRNA expression of interferon alpha 2 (*IFNA2*), interferon beta 1 (*IFNB1*), interferon lambda 1 (*IFNL1*), and interferon lambda 2 (*IFNL2*), which are markers of type I or III interferon responses (56), could not be detected by qRT-PCR in infected hBECs.

### Internalization of *T. marneffei* by hBECs

**Fig 6:**
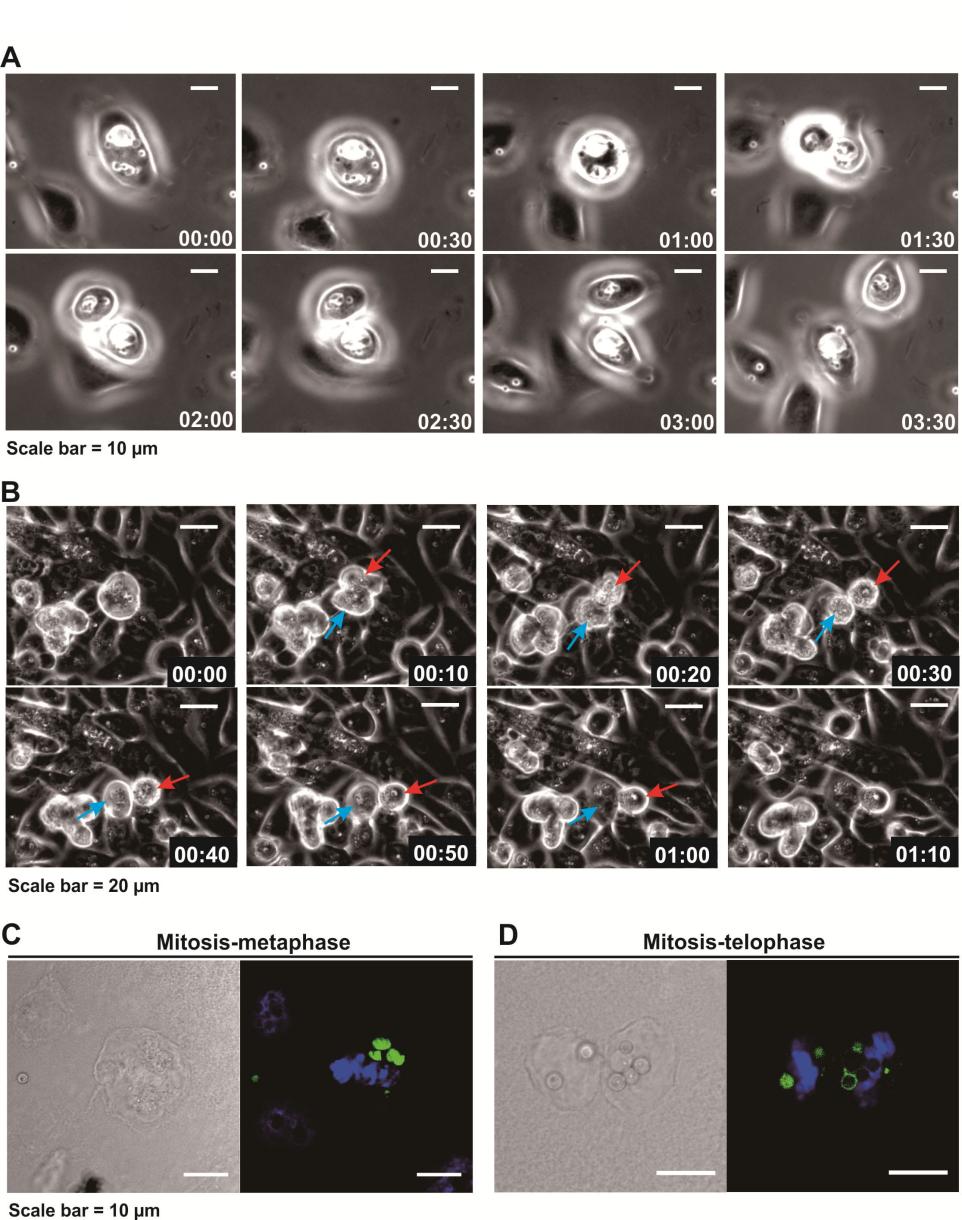
Image analysis of hBECs and *Talaromyces marneffei* interactions. (**A**) Using live cell imaging, the process of cell division was observed in *T. marneffei*-infected hBECs as shown in high (**A**) and low (**B**) powers. Time lapse is indicated in a format of hh:mm at the bottom right. *T. marneffei*-infected hBECs at (**C**) metaphase and (**D**) telophase were indicated in confocal images.

To investigate the interactions between hBECs and *T. marneffei*, we first generated a GFP-tagged *T. marneffei* strain (PM1-GFP). Growth of this genetically modified fluorescent strain at both 25°C and 37°C showed similar morphologies and growth rates to its parental strain (PM1), respectively (Fig. S2B and C); and this fluorescent strain was then used for subsequent confocal microscopy analyses. Typical stacking of optical sections (z-series) showed that GFP signals of cell surface-attached conidia and internalized conidia were detected from different sections. Visualization of the GFP signals revealed that most of the signals were detected from inside of the cells, which was in line with the expected subcellular localization (Fig. 5; Fig. S3; Movie S1), indicating that fungal conidia were not only attached to the surface of hBECs, but also present inside the host cells. The results suggested that hBECs might play a role in fungal recognition and engulfment. To acquire a more in-depth physiological understanding on the interactions between hBECs and *T. marneffei*, live cell imaging for a continuous 24-h period was performed. It was observed that internalized conidia appeared to be localized inside hBECs via unknown pathways (Fig. 6A and B; Fig. S4; Movie S2 and S3). Moreover, the process of cell division was widely observed in the infected hBECs (Fig. 6A and B; Movie S2 and S3), which was confirmed by confocal microscopy (Fig. 6C and D and Fig. S5 and S6).

### Survival of *T. marneffei* in hBECs

**Fig 7:**
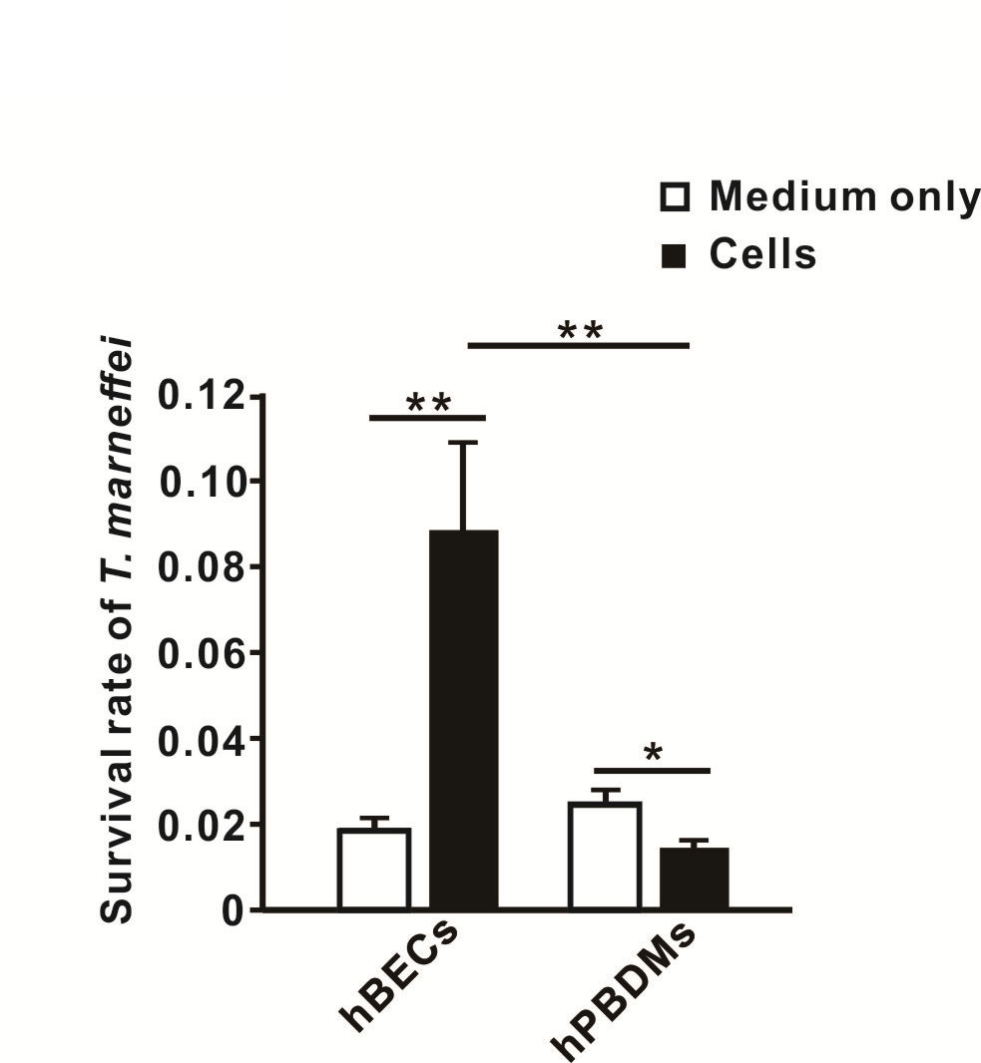
Survival rates of *Talaromyces marneffei* in hBECs and hPBDMs. The survival rate of *T. marneffei* in hBECs was markedly higher than those in medium and in hPBDMs (both *P <* 0.01). Data are presented as mean ± SD and comparisons were analyzed using unpaired *t*-test (two-tailed). *, *P* < 0.05; **, *P* < 0.01.

Next, we sought to determine the fates of internalized *T. marneffei* conidia in the immunologically silent hBECs. Notably, the culture medium was supplemented with 240 U/mL of nystatin at 24 h post-infection in order to inhibit the extracellular growth of *T. marneffei*, and the recovery of internalized *T. marneffei* from the host was determined by sub-culturing co-cultured cell-pathogen lysates onto agar plates. It was found that the lysates from hBEC*-T. marneffei* co-culture yielded a higher fungal recovery rate than those of hPBDM*-T. marneffei* co-culture (*P <* 0.01) (Fig. 7; Table S3).

## DISCUSSION

We established an *in vitro* human macrophage system for the study of innate immune response to *T. marneffei* infection. In previous host-pathogen interaction studies on *T. marneffei* that employed murine macrophages, very often, pre-treatment of the macrophages with stimulants such as IFN-γ, LPS, and/or phorbol 12-myristate 13-acetate (PMA) was performed prior to the experiments (13, 22, 24 – 26, 28 – 33). However, such stimulation was non-physiological and did not represent the situation in human *T. marneffei* infection. Therefore, in this study, we first aimed to develop a cell infection model using human macrophages which could better mimic the *in vivo* situations for the characterization of innate immune response against talaromycosis in humans. Although studies in the last decade have shown that the majority of tissue-resident macrophages (e.g., alveolar macrophages in the lungs) are established prenatally from the yolk sac or fetal liver during embryonic development and are able to self-maintain locally with minimal replacement by circulating monocytes under homeostatic conditions (57), a recent mouse model study revealed that the transcriptome of embryonic-derived lung-resident alveolar macrophages and that of postnatal monocyte-derived alveolar macrophages exhibited >98% correlation and alveolar macrophages of the two origins displayed no observable difference in phagocytic and immunomodulatory abilities (58). Given that monocytes from donated blood are more readily available, there are well-established protocols for the isolation of peripheral blood monocytes. Circulating monocytes are recruited into the lungs during inflammation/infection (59) and embryonic and postnatal monocyte-derived alveolar macrophages are highly similar in their transcriptomes and phenotypes (58). In the current study, we employed the use of hPBDMs for the elucidation of the host innate immune response to *T. marneffei* infection.

In the first part of the present study, we showed that *TNF* and, more markedly, *CXCL8* transcriptions in hPBDMs were induced at 24 h post-*T. marneffei* infection without IFN-γ and/or LPS stimulation (Fig. 1A). Such induction was only present when autologous plasma was added to the hPBDMs, suggesting that certain humoral factors in the plasma were essential for the defense mechanism, especially on cytokine and chemokine stimulation, against *T. marneffei* infection. Indeed, in the absence of autologous plasma and hence these humoral factors, transcriptions of both *TNF* and *CXCL8* in hPBDMs were significantly suppressed at 24 h post-*T. marneffei* infection (Fig. 1B). This suggested that *T. marneffei*, via unknown mechanisms, might be able to inhibit immune response by hPBMDs although such suppression could be overcome by the immune induction mediated by those certain blood-circulating humoral factors. On the other hand, transcriptions of both *TNF* and *CXCL8* were not induced in two murine macrophage cell lines (J774A.1 and RAW264.7) (Fig. 1C and D), indicating that such murine systems that were often used to study *T. marneffei* infections (12, 15, 17, 18, 34), although already supplemented with FBS, cannot reflect the situation in human. Further identification of such factors in human plasma will reveal the underlying mechanism of innate immune response during early infection of *T. marneffei*.

Human macrophages are activated to the M1-like phenotypes upon *T. marneffei* infection. Macrophages are extremely plastic and therefore can possess heterogeneous phenotypes (60, 61). Early studies classified activated macrophages by phenotypes using a dichotomous system as M1 (classically activated) and M2 (alternatively activated) macrophages (60). Broadly speaking, M1 macrophages are activated in an inflammatory environment by Toll-like receptor and interferon signaling, are pro-inflammatory, and possess antimicrobial activities (61). On the other hand, M2 macrophages are associated with T helper cell type 2 response and are mainly anti-inflammatory (61). The activation of macrophages into the M1/M2 phenotypes are traditionally termed “macrophage polarization”. However, studies in recent decades have recognized that phenotypes of activated macrophages indeed could not be simply represented using a binary system (M1/M2 polarization) only (62). Instead, there exist a spectrum of activated macrophage phenotypes; and so a multidimensional model integrating the microenvironmental signals to which these macrophages are exposed is needed for the description of their activation statuses and the characterization of their specific functions (62). Nonetheless, standardization of the nomenclature for macrophage activation is still underway (63), while the terms M1/M2 (-like) activation/polarization are still frequently used. Although macrophage activation has largely been studied using mouse models, one previous study by Martinez et al. attempted to characterize the transcriptomic changes during human monocyte-to-macrophage differentiation as well as upon macrophage activation (M1/M2 polarization) (49). It was found that a panel of genes (*n* ~ 100) were strictly associated with macrophage activation (differentially expressed in M1-like macrophages versus M2-like macrophages) and these genes could be grouped into seven functional categories, namely membrane receptors, cytokines and chemokines, apoptosis-related genes, solute carriers, enzymes, extracellular mediators as well as DNA-binding factors (49). In the present study, using the hPBMC model established where the cells were cultured in the presence of human autologous plasma, we characterized the change in transcriptomic profiles of hPBMCs during the early stage of *T. marneffei* infection, with an aim to understand how macrophages are activated. Of the 452 genes differentially expressed in hPBDMs upon *T. marneffei* infection identified in this study, 25 were associated with macrophage activation when compared with Martinez et al.’s findings (49). Notably, all these 25 DEGs associated with macrophage activation were found to exhibit the same expression change pattern as M1-like macrophages, suggesting that *T. marneffei*-infected macrophages might, in general, undergo M1-like activation during the early phase of infection.

In particular, mRNA expression of the chemokine genes *CXCL10*, *CCL5* (C-C motif chemokine ligand 5), and *CCL20* (C-C motif chemokine ligand 20) was significantly up-regulated and this could help recruit other immune cells such as lymphocytes and dendritic cells to the infection sites for pathogen removal. The mRNA expression of *TNF* was also significantly up-regulated, favoring the induction of inflammation. In addition, activation of macrophages upon *T. marneffei* infection was associated with changes in the expressions of extracellular mediators as well as membrane receptors (Table 1). In the literature, a few previous studies have attempted to characterize macrophage activation during *T. marneffei* infection, albeit using an experimental mouse model or a murine cell line. In 2017, Dai et al. showed that alveolar macrophages in *T. marneffei*-infected mice were activated toward both the M1-like and M2a-like phenotypes at 2 weeks post-infection, after which the activations began to resolve (23). Another recent study by Lu et al., through the assessment of the change in cell surface marker expressions using a murine macrophage cell line (RAW264.7), demonstrated that both resting (M0-like) and activated M2-like macrophages could be activated or cross-conversed to the M1-like phenotypes upon *T. marneffei* infection under IFN-γ stimulation (27), respectively. However, the first study only investigated the activation statuses of macrophages at 2 weeks and 4 weeks post-infection (23), and these may not represent the situation during the early phase of *T. marneffei* infection. Moreover, both these two studies only employed murine models and their assessment on macrophage activation relied partly on the use of murine macrophage activation markers inducible nitric oxide synthase (for M1-like phenotype) and arginase 1 (Arg1, for M2-like phenotype) (23, 27). However, given the differences between human and murine macrophages, whether macrophage activation examined based on these markers can be translated to the situations in humans is not known (63, 64). Furthermore, the use of Arg1 as the marker for M2-like phenotype may also not be appropriate in all cases (64). Another more recent study suggested that *T. marneffei* infection might lead to an M2 polarization in human macrophages (36). However, this finding was largely based on PMA (M0)-, PMA/LPS/IFN-γ (M1-like)-, or PMA/IL-4 (M2-like)-stimulated THP-1 cell model and human peripheral blood mononuclear cells (PBMCs) interacting with a high concentration of spores (MOI = 10) (36). The application of PMA-differentiated THP-1 macrophages raised concerns regarding its ability to fully replicate the range of responses observed in primary monocyte-derived macrophages when exposed to activating stimuli (65). Nevertheless, in the present study, we aimed to gain a better understanding toward the natural infection of *T. marneffei* that presumably happens in human lungs. PBMCs were isolated and subjected to a 2-week attachment step in order to facilitate the selection of macrophages before being allowed to interact with *T. marneffei*; and we found that vigorous immune response and M1 polarization can be induced in these hPBDMs upon *T. marneffei* infection. Our results might potentially elucidate why *T. marneffei* only impacts individuals with weakened immune systems, leading to severe illness. Therefore, to better understand the innate immune response against *T. marneffei* infection in humans, a cell culture model of human macrophages is needed and our current study provided the first evidence showing the gross transcriptional changes toward M1-like activation in human macrophages during the first 48 h of *T. marneffei* infection.

In contrast to hPBDMs, which vigorous immune response was triggered upon encountering *T. marneffei*, cytokine response was not induced in hBECs during *T. marneffei* infection. It has been shown previously (53, 66, 67) and in the present study that transcriptions of *TNF*, *CXCL8,* and *CXCL10* in hBECs were induced upon *A. fumigatus* infection (Fig. 5A), which could help recruit neutrophils, phagocytes, and/or other immune cells to the sites of infection for fungal clearance. On the other hand, transcriptions of such cytokine genes as well as *IFNA2*, *IFNB1*, *IL1B*, *IL6*, *IFNL1,* and *IFNL2* in hBECs were not induced during *T. marneffei* infection (Fig. 5A), despite the demonstration of the presence of live conidia inside hBECs (Fig. 5B). This implicated that the affected hBECs were unlikely to illicit any innate immune response, different from the situation observed for hPBMCs, which the expressions of *TNF*, *CXCL8*, *CXCL10* as well as other immunoregulatory genes including *SOD2*, *CCL3*, *STAT1*, *CLEC4E*, *IL1B*, *IER3*, *PIK3R2,* and *C3* were significantly increased post-infection (Fig. 2 and 4A). Moreover, confocal microscopy and live cell imaging demonstrated that the infected hBECs were still able to undergo cell division, where cells at various cell cycle stages such as metaphase and telophase as well as the process of cell doubling were observed (Fig. 6; Movie S2 and S3), implying that the infection did not severely affect cellular physiology. Intracellular survival assays showed that *T. marneffei* survived better in infected hBECs than in hPBDMs (Fig. 7B), suggesting an ineffective clearance of *T. marneffei* by hBECs. As a whole, the results of the present study illustrated a potential role of hBECs to serve as reservoir cells, inside which *T. marneffei* hides so as to evade immunosurveillance by macrophages or other professional phagocytic cells. More importantly, the ability of *T. marneffei* to grow as individual yeast-like cells *in vivo* allows the fungus to parasitize inside hBECs without forming extracellular hyphae which are subjected to immunosurveillance. As such, we envisage that after being ingested by hBECs, the growth of *T. marneffei* would attain a homeostatic status. While *T. marneffei* hiding inside hBECs are able to replicate intracellularly, excessive *T. marneffei* cells would be released out of the hBECs and get killed by phagocytic cells. When the immunity of the host is weakened later (e.g., due to HIV infection) and fails to eliminate these excessive *T. marneffei* cells, infection would develop and thus lead to clinical manifestations. This might also explain the long incubation period (up to 11 years) of talaromycosis especially for travel-related cases (68) where although the patients acquired *T. marneffei* as early as their travels to endemic areas, infection only developed years later.

## Data Availability

The RNA–Seq raw data have been deposited in the National Center for Biotechnology Information (NCBI) Sequence Read Archive (SRA) BioProject PRJNA564361.

## SUPPLEMENTAL MATERIAL

Supplemental figures msphere.00258-22-s0001.pdf Fig. S1 to S6.

Legends msphere.00258-22-s0002.pdf Legends for supplemental files.

Table S1 msphere.00258-22-s0003.pdf Primers used in this study.

Table S2 msphere.00258-22-s0004.xlsx Detailed information regarding the 452 differentially expressed genes identified in this study.

Table S3 msphere.00258-22-s0005.xlsx CFU count of *Talaromyces marneffei* in hBECs and hPBDMs.

Movie S1 msphere.00258-22-s0006.mp4 Three-dimensional view of Talaromyces marneffei strain PM1-GFP conidia in human bronchial epithelial cells (hBECs).

Movie S2 msphere.00258-22-s0007.mp4 Interaction of human bronchial epithelial cells (hBECs) and Talaromyces marneffei conidia at low magnification (20×).

Movie S3 msphere.00258-22-s0008.mp4 Interaction of human bronchial epithelial cells (hBECs) and Talaromyces marneffei conidia at higher magnification (40×).

ASM does not own the copyrights to Supplemental Material that may be linked to, or accessed through, an article. The authors have granted ASM a non-exclusive, world-wide license to publish the Supplemental Material files. Please contact the corresponding author directly for reuse.
