# Supplementary material for: Differential innate immune responses of human macrophages and bronchial epithelial cells against Talaromyces marneffei
Source: mSphere. 2023 Sep 11;8(5):e00258-22. doi: 10.1128/msphere.00258-22 (PMC10597461; doi:10.1128/msphere.00258-22)
Supplement: Supplemental figures — Fig. S1 to S6. [file msphere.00258-22-s0001.pdf]

Figure S1

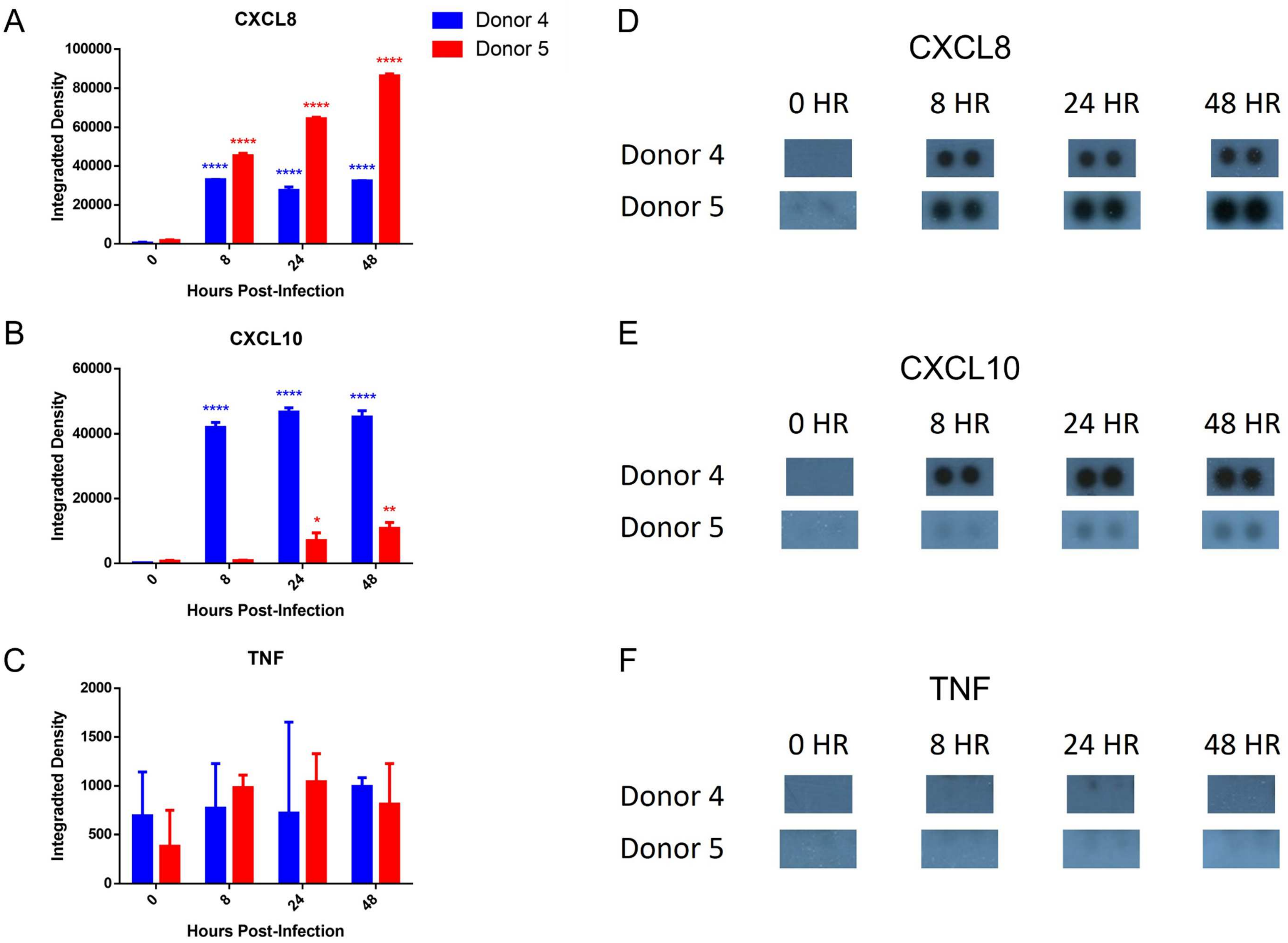
A
CXCL8
Donor 4
Donor 5
Integradted Density
100000
80000
60000
40000
20000
0
****
0
8
24
48
Hours Post-Infection
B
CXCL10
60000
40000
20000
*
**
C
TNF
2000
1500
1000
500
D
CXCL8
0 HR
8 HR
24 HR
48 HR
E
CXCL10
F
TNF

Figure S2

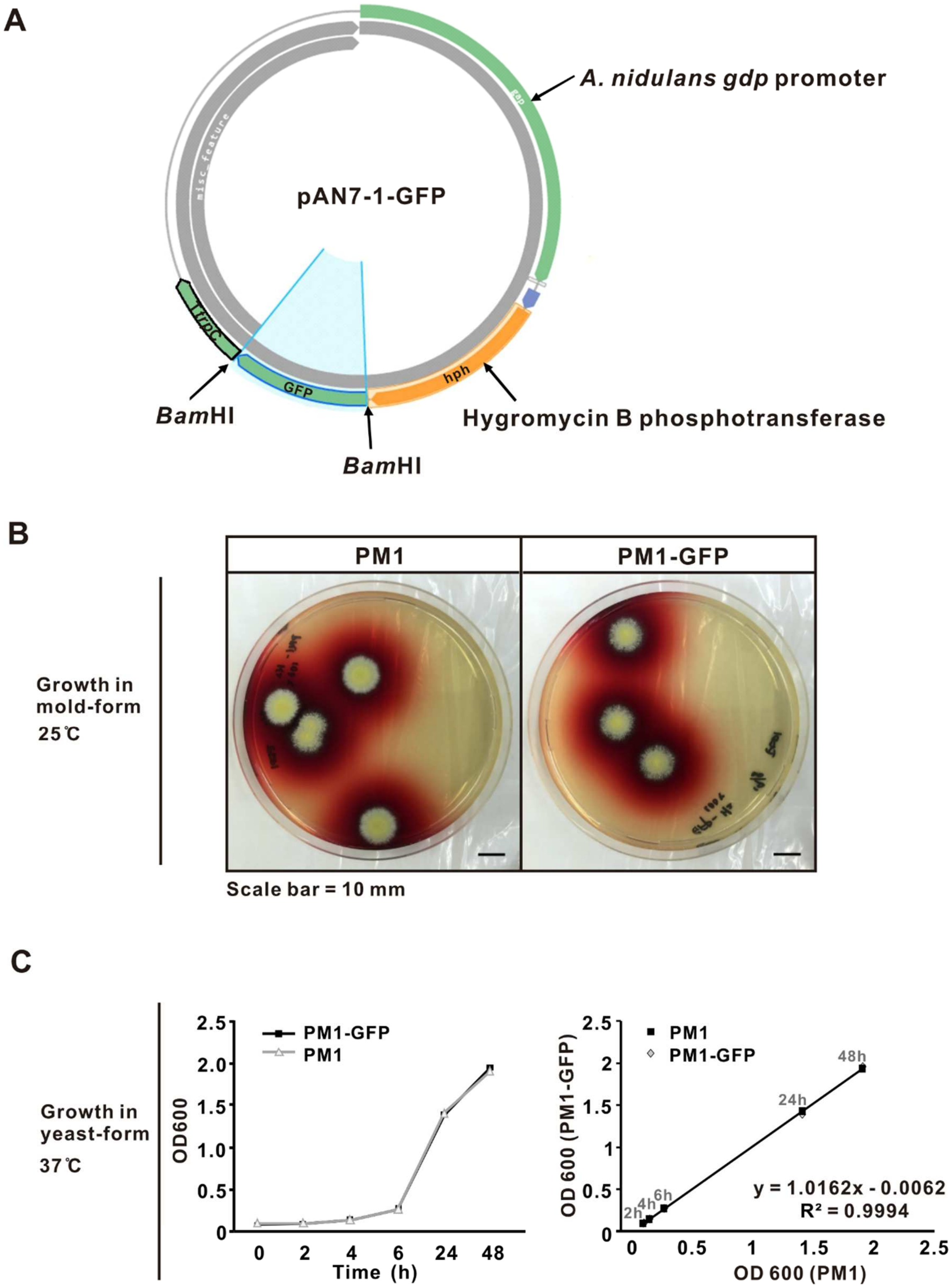

A
A. nidulans gdp promoter
pAN7-1-GFP
TtrpC
GFP
hph
BamHI
BamHI
Hygromycin B phosphotransferase
B
PM1
PM1-GFP
Growth in mold-form 25℃
Scale bar = 10 mm
C
Growth in yeast-form 37℃
PM1-GFP
PM1
OD600
Time (h)
PM1
PM1-GFP
OD 600 (PM1-GFP)
OD 600 (PM1)
y = 1.0162x - 0.0062
R² = 0.9994
2h
4h
6h
24h
48h

Figure S3

A

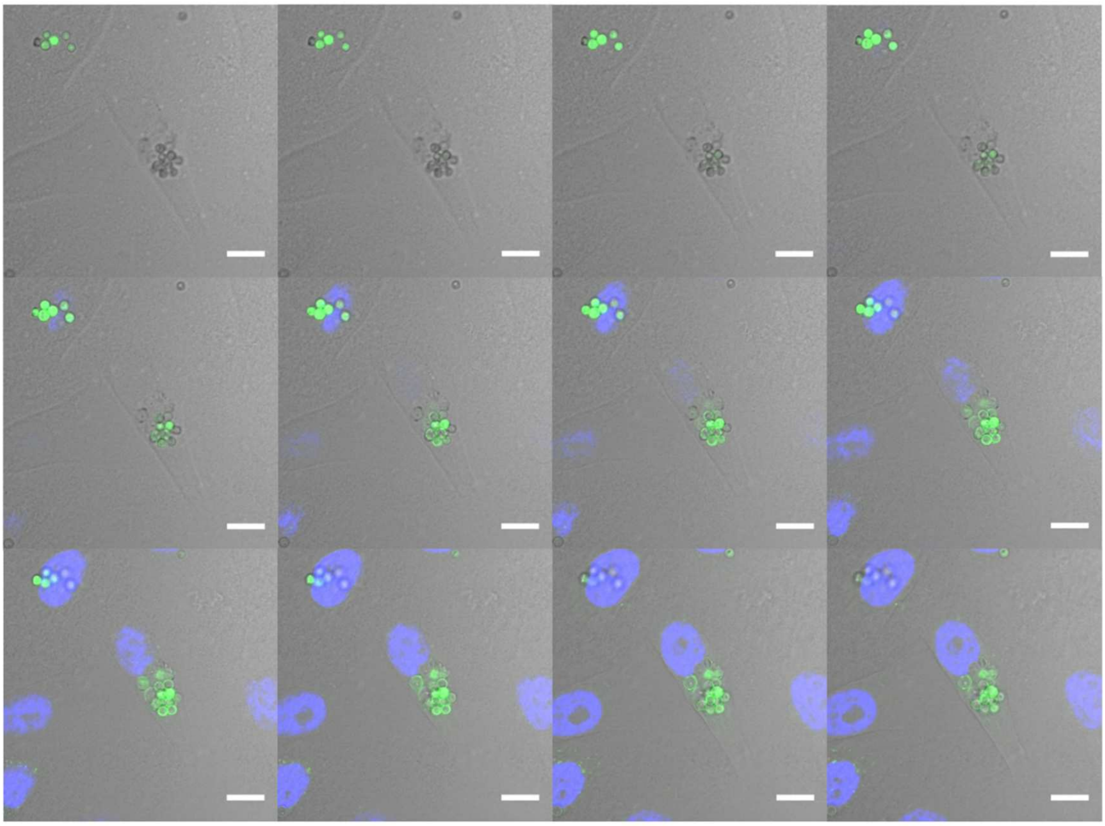

Scale bar = 10 μm

B

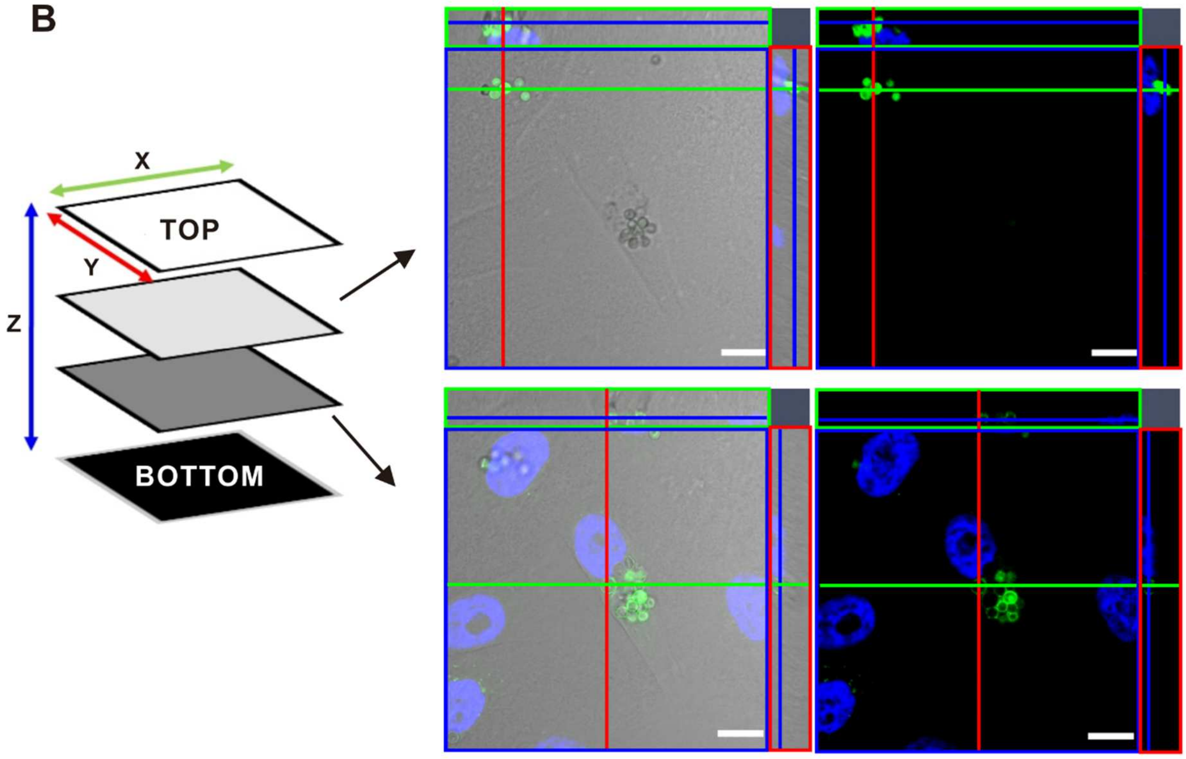

Scale bar = 10 μm

Figure S4

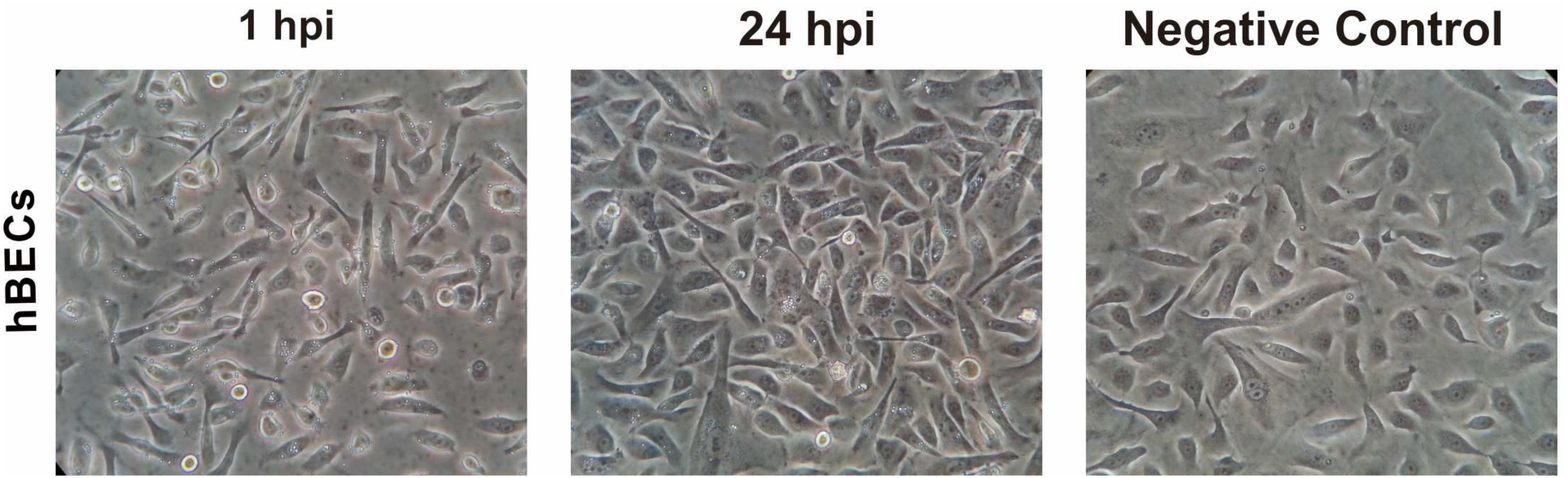

Figure S5

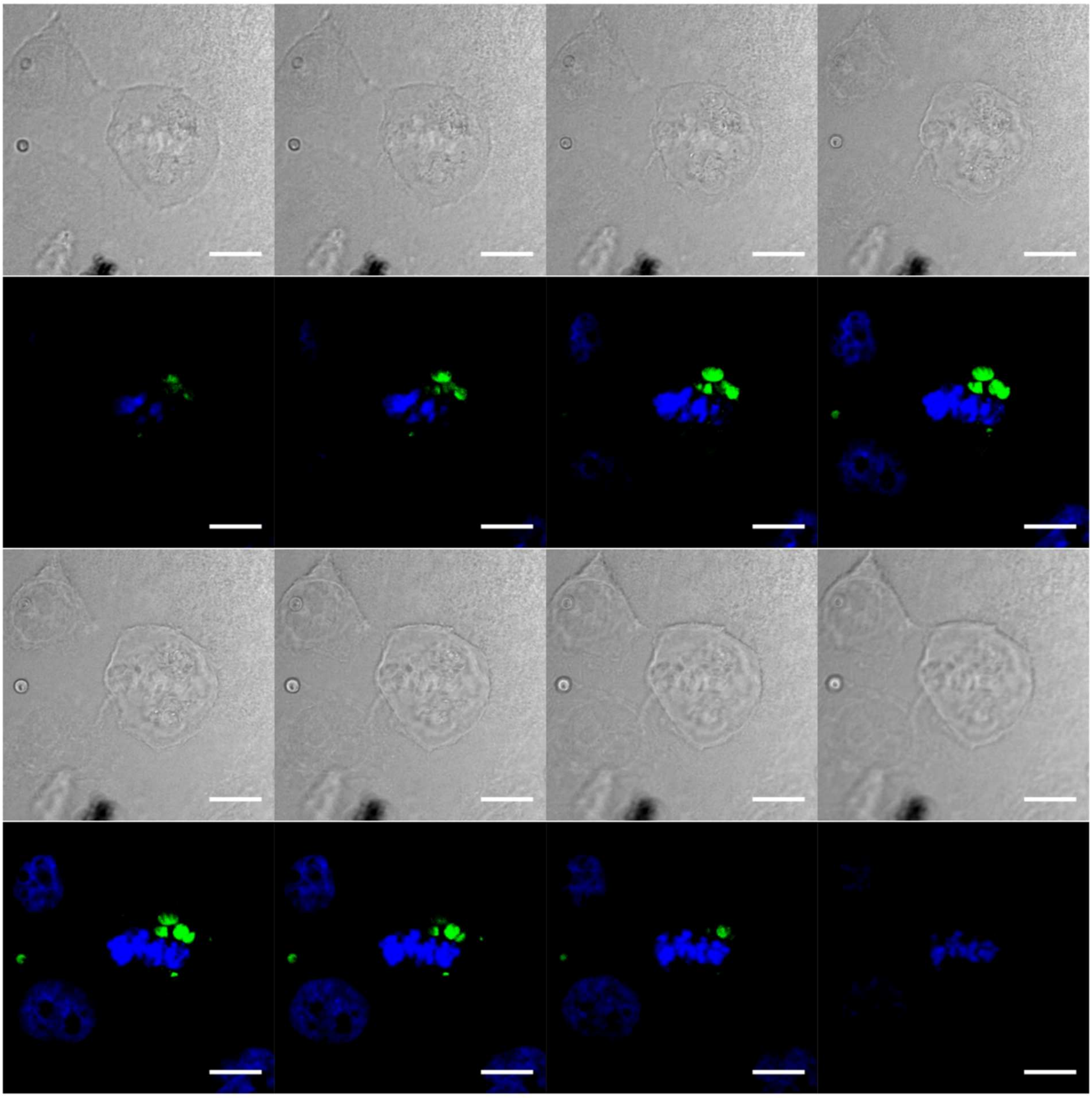

Scale bar = 10 μm

Figure S6

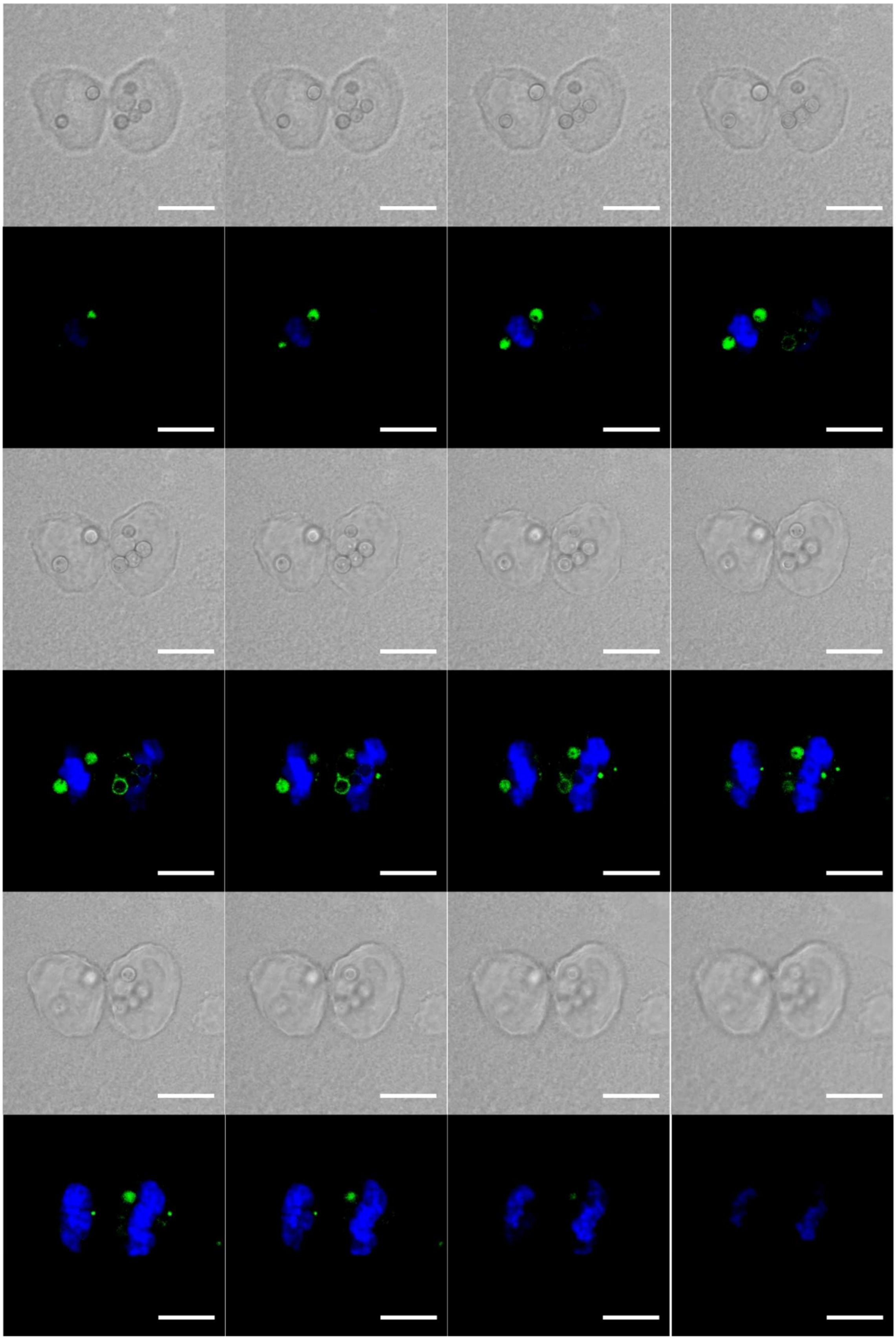

Scale bar = 10 μm
