# Supplementary material for: Differential innate immune responses of human macrophages and bronchial epithelial cells against Talaromyces marneffei
Source: mSphere. 2023 Sep 11;8(5):e00258-22. doi: 10.1128/msphere.00258-22 (PMC10597461; doi:10.1128/msphere.00258-22)
Supplement: Legends — for supplemental files. [file msphere.00258-22-s0002.pdf]

LEGENDS FOR SUPPLEMENTARY MATERIALS

Figure S1. Changes of cytokine protein expression in human peripheral blood-derived macrophages (hPBDMs) upon *Talaromyces marneffei* infections. Relative protein expressions of CXCL8 and CXCL10 in hPBDMs infected with *T. marneffei* were significantly induced after 8, 24 and 48 h post-infection while the protein expression of TNF in hPBDMs infected with *T. marneffei* infection did not change significantly after infection. The infection experiment was conducted in duplicate using blood cells isolated from three independent donors. The relative protein expression levels of CXCL8, CXCL10 and TNF were detected using the Proteome Profiler Human XL Cytokine Array Kit. Data are presented as mean ± SD and comparisons were analysed using one-way ANOVA. *, $p < 0.05$; **, $p < 0.01$; ****$p < 0.0001$.

Figure S2. Green fluorescent protein (GFP)-tagged *Talaromyces marneffei* strain. (A) Plasmid map of pAN7-1-GFP. GFP protein sequence, which was amplified using pEGFP-C1 as a template, was cloned into the plasmid pAN7-1 at the *Bam*HI restriction site for transformation into *T. marneffei* strain PM1. (B) The morphology of *T. marneffei* PM1-GFP in filamentous form resembled that of its parental strain, PM1. (C) The growth kinetics of PM1-GFP yeasts were further determined by recording the optical density at 600 nm (OD_{600}) in the liquid medium yeast peptone dextrose (YPD) broth. Linear regression analysis revealed that the growth of *T. marneffei* PM1-GFP in yeast form was similar to that of its parental strain, PM1 ($R^2 = 0.9994$).

Figure S3. The extracellular and intracellular distribution of conidia in human bronchial epithelial cells (hBECs). (A) Serial Z-stack images indicating the

distribution of *T. marneffei* conidia. (B) The extracellular and intracellular distribution of conidia in orthographic diagrams with different x, y and z plane views.

Figure S4. Morphological appearance of human bronchial epithelial cells (hBECs) at 1 and 24 h post-infection. Clear morphological changes with plenty of vesicle-structures could be observed within hBECs after 24 h post-infection.

Figure S5. Serial Z-stack images indicating the distribution of conidia within a human bronchial epithelial cell (hBEC) in mitosis-metaphase.

Figure S6. Serial Z-stack images indicating the distribution of conidia within a human bronchial epithelial cell (hBEC) in mitosis-telophase.

Movie S1. Three-dimensional view of *Talaromyces marneffei* strain PM1-GFP conidia in human bronchial epithelial cells (hBECs).

Movie S2. Interaction of human bronchial epithelial cells (hBECs) and *Talaromyces marneffei* conidia at low magnification (20×).

Movie S3. Interaction of human bronchial epithelial cells (hBECs) and *Talaromyces marneffei* conidia at higher magnification (40×).

Table S1. Primers used in this study.

Table S2. Detailed information regarding the 452 differentially expressed genes identified in this study.

Table S3. Colony-forming unit (CFU) count of *Talaromyces marneffei* in human bronchial epithelial cells (hBECs) and human peripheral blood-derived macrophages (hPBDMs).
