# Supplementary material for: Differential innate immune responses of human macrophages and bronchial epithelial cells against Talaromyces marneffei
Source: mSphere. 2023 Sep 11;8(5):e00258-22. doi: 10.1128/msphere.00258-22 (PMC10597461; doi:10.1128/msphere.00258-22)
Supplement: Table S1 — Primers used in this study. [file msphere.00258-22-s0003.pdf]

Table S1. PCR primers used in the present study.

Primer	Target gene	Complementarity[a]	Sequences (5′ to 3′)
LPW31624	EGFP	F	CGGGATCCATGGTGAGCAAGGGC[b]
LPW31625	EGFP	R	GTGGATCCCGGGCCCGCGGTAC[b]
LPW34513	Mouse TNFα	F	CATCTTCTCAAAATTCGAGTGACAA
LPW34514	Mouse TNFα	R	TGGGAGTAGACAAGGTACAACCC
LPW34515	Mouse IL8	F	GAAGTCATAGCCACTCTCAAGG
LPW34516	Mouse IL8	R	TTCCGTTGAGGGACAGCA
LPW34517	Mouse IP10	F	ATCAGCACCATGAACCCAAG
LPW34518	Mouse IP10	R	GTGGCAATGATCTCAACACG
LPW34519	Mouse actin	F	AGAGGGAAATCGTGCGTGAC
LPW34520	Mouse actin	R	CAATAGTGATGACCTGGCCGT
LPW34521	TNFα	F	CTGCTGCACTTTGGAGTGAT
LPW34522	TNFα	R	AGATGATCTGACTGCCTGGG
LPW34523	IL8	F	CTGATTTCTGCAGCTCTGTG
LPW34524	IL8	R	GGGTGGAAAGGTTTGGAGTATG
LPW34525	IP10	F	CCATTCTGATTTGCTGCCTTAT
LPW34526	IP10	R	TTTCCTTGCTAACTGCTTTCAGTA
LPW34527	GAPDH	F	GAGTCAACGGATTTGGTCGT
LPW34528	GAPDH	R	TTGATTTTGGAGGGATCTCG
LPW35393	SOD2	F	GCTCCGGTTTTGGGGTATCTG
LPW35394	SOD2	R	GCGTTGATGTGAGGTTCCAG
LPW35395	CCL3	F	AGTTCTCTGCATCACTTGCTG
LPW35396	CCL3	R	CGGCTTCGCTTGGTTAGGAA
LPW35397	STAT1	F	ATCAGGCTCAGTCGGGGAATA
LPW35398	STAT1	R	TGGTCTCGTGTTCTCTGTTCT
LPW35399	CLEC4E	F	CTGAAACACAATGCACAGAGAGA
LPW35400	CLEC4E	R	AAAGATGCGAAATGTCACAACAC
LPW35401	IL1B	F	AGCTACGAATCTCCGACCAC
LPW35402	IL1B	R	CGTTATCCCATGTGTCGAAGAA
LPW35405	IER3	F	CAGCCGCAGGGTTCTCTAC
LPW35406	IER3	R	GATCTGGCAGAAGACGATGGT
LPW35407	PIK3R2	F	TCACCTTCTGCTCCGTTGTG
LPW35408	PIK3R2	R	GGAGGTCCGTGTGTACTCTTC
LPW35409	CD9	F	TTCCTCTTGGTGATATTCGCCA
LPW35410	CD9	R	AGTTCAACGCATAGTGGATGG
LPW35411	AIF1	F	ATGAGCCAAACCAGGGATTTAC

LPW35412	AIF1	R	GGGATCGTCTAGGAATTGCTTGT
LPW35413	CD180	F	AACCTAAGCCTGAACTTCAATGG
LPW35414	CD180	R	GCCAGAGAGACTGAGTAGTAGAG
LPW35415	TLR4	F	AGACCTGTCCCTGAACCCTAT
LPW35416	TLR4	R	CGATGGACTTCTAAACCAGCCA
LPW35904	FCN1	F	GGGCAGTGCGGGTAATTCTC
LPW35905	FCN1	R	GAAGCATGACAGTCGGCGTA
LPW35906	C3	F	GGGGAGTCCCATGTACTCTATC
LPW35907	C3	R	GGAAGTCGTGGACAGTAACAG
LPW35908	C3AR1	F	AAGCCAATCTGGTGTCAGAATC
LPW35909	C3AR1	R	CAGGAATGCACATCACAAAAGC
LPW35910	C1QA	F	TCTGCACTGTACCCGGCTA
LPW35911	C1QA	R	CCCTGGTAAATGTGACCCTTTT
LPW35912	C1QC	F	AGGATGGGTACGACGGACTG
LPW35913	C1QC	R	GTAAGCCGGGTTCTCCCTTC
LPW35914	VSIG4	F	GGGGCACCTAACAGTGGAC
LPW35915	VSIG4	R	GTCTGAGCCACGTTGTACCAG

[a]F, forward primer; R, reverse primer

[b]GGATCC, *Bam*HI restriction site
